# Peer Learning in Medical and Health Professions Education: Evidence, Design Principles, and Pedagogical Reflection

**DOI:** 10.1177/23821205261486310

**Published:** 2026-08-31

**Authors:** Adrian Elmi-Terander

**Affiliations:** 1 Department of Clinical Neuroscience, 27106 Karolinska Institutet, Stockholm, Sweden

**Keywords:** peer learning, peer assisted learning, medical education, feedback, evaluation, clinical reasoning

## Abstract

Peer learning is a collaborative approach grounded in social constructivist theory, emphasizing that knowledge is co-created through interaction. In medical education, it promotes active engagement, critical thinking, and professional identity formation. Within the author’s research group, PhD students in the field of neurosurgery and medical students working on their undergraduate medical research projects, benefit from structured peer learning by sharing expertise, discussing challenges, and providing feedback. This aligns with communities of practice theory, where learning occurs through participation in a shared domain, and with Topping’s framework highlighting peer tutoring as a reciprocal process that enhances cognitive and social development. Recent studies demonstrate that peer assisted learning improves clinical knowledge, practical skills acquisition, and learner engagement compared with traditional teaching approaches. These effects appear particularly strong in clinical and applied contexts, with evidence suggesting retention beyond the immediate learning period and improved transfer to performance. Integrating peer learning as a deliberate strategy strengthens academic outcomes and fosters lifelong learning skills. The Kirkpatrick framework can be used to evaluate the design, implementation, and outcomes of peer learning (PL) interventions.

In line with these traditions, recent work in Academic Medicine has clarified mechanisms through which longitudinal near-peer learning operates—structured preparation, dynamic role-shifting during joint encounters, and post-encounter debriefing within trusted relationships—offering concrete practices that can be translated into the proposed setting.

## Introduction

Peer learning is defined as students learning with and from other students through structured interaction, feedback, and shared problem-solving. It has strong theoretical background in social constructivism and sociocognitive theories. Vygotsky’s proposition that knowledge is co-constructed through interaction underpins why peers can be powerful resources, particularly in environments where guidance from more knowledgeable peers enhances performance.^1^ Bandura’s social learning theory further explains how modeling, vicarious reinforcement, and self-efficacy development contribute to learning gains when students observe and emulate peers’ strategies and performance.^2^

More recent literature has extended these theoretical foundations by identifying specific mechanisms through which peer learning occurs. Contemporary work demonstrates that peer assisted learning fosters elaboration through explanation and discussion, co-construction of knowledge through interaction, and iterative feedback processes that enable learners to refine understanding in real time.^3,4^ Longitudinal near peer learning environments have been shown to operate through structured preparation, dynamic role shifting during interaction, and structured debriefing, enabling feedback that is closely aligned with learners’ developmental level and enhancing clinical reasoning performance.^5^ Evidence from longitudinal educational models further suggests that continuity with peers, supervisors, and learning activities enhances learner engagement, mentoring relationships, and educational outcomes.^6^

The pedagogical literature mentions multiple forms of peer learning, like peer tutoring (including near-peer- and peer to peer learning), cooperative learning, peer-assisted learning (PAL), and peer assessment and feedback. In his review of 2005, Topping synthesized decades of evidence showing cognitive, metacognitive, and social benefits for both learners and student-tutors, and provided design parameters like matching, training, structure, and assessment, that influence effectiveness.^7^ Others articulate how reciprocal peer learning can be deliberately embedded into course design and assessment at scale, turning informal practices into systematic strategies aligned with higher education goals.^8^ In her book “Learning Together”, Falchikov offered practical guidance and a theoretical synthesis for peer tutoring schemes, including typical problems and solutions in implementation.^9^ Ten cate and Durning describe what theory can offer to understand dynamics and psychology of peer teaching.^10^

In medical education, peer teaching and PAL are widely adopted and supported by empirical research. Ten Cate and Durning, surveyed the 2006 medical education literature, outlining dimensional frameworks (educational distance, formality, group size) and twelve reasons, from efficiency to professional identity formation, arguing for the routine use of peer teaching in medical curricula.^11^ Lockspeiser et al, studying medical students’ experiences, demonstrated the value of social and cognitive congruence. They found that near-peers connect to learners’ current knowledge, language, and struggles, enhancing comprehension and motivation.^12^ Several systematic reviews corroborate comparable outcomes of peer teaching when contrasted with faculty-led instruction in selected contexts and reveal additional gains for student-teachers.^4,13-15^ More recent evidence suggests that participation as a peer teacher further contributes to development of professional identity, educational skills, leadership behaviors, and self-efficacy among medical students.^16^ Secomb’s review of clinical education shows benefits across psychomotor and cognitive domains like confidence and skill acquisition, while also noting potential risks such as compatibility issues and reduced individualized instructor time if poorly designed.^14^

While early literature established the benefits of peer learning, more recent systematic reviews and meta-analyses provide stronger empirical support. Peer assisted learning has been shown to improve knowledge acquisition and clinical performance compared with traditional instructional approaches, with particularly strong effects in applied and skills-based domains.^17,18^ Furthermore, a recent large scale meta-analysis of randomized controlled trials demonstrates that peer assisted learning improves not only knowledge outcomes but also skills acquisition and learner satisfaction, reinforcing its role as an effective strategy in contemporary medical education.^19^

In a recent systematic review and meta-analysis of randomized trials, Brierley et al, reported that medical students taught via PAL achieved significantly higher academic performance than controls. Effects were larger among clinical students than preclinical students, and for practical skills teaching, compared with theory, where effects were not significant. Notably, PAL advantages persisted in assessments conducted more than four weeks after course completion, suggesting medium-term retention benefits.^15^

Beyond immediate performance, peer learning contributes to communities of practice in which learners advance from peripheral to more central participation through shared activity and mutual engagement.^20^ Such participation may also contribute to professional identity formation, which is increasingly recognized as a central outcome of medical education and a process shaped through social interaction, workplace participation, and longitudinal learning relationships.^21^ These communities function as living curricula that socialize learners into disciplinary norms and collaborative problem-solving, which is central for research-intensive fields like neurosurgery. Peer assessment can be harnessed to improve academic writing, presentation, and research design. Topping’s synthesis on peer assessment emphasizes elaborated peer feedback, reliability under certain conditions, and the developmental value of judging others’ work.^7^ Works by Gielen et al and Carless et al, have expanded typologies, clarified goals, and identified feedback characteristics that enhance learning.^22-24^ Recent work has also highlighted the importance of feedback literacy within peer learning environments.^24^ Feedback literacy refers to learners’ ability to interpret, evaluate, and use feedback effectively, while evaluative judgement describes the capacity to assess the quality of one’s own and others’ work. Both are essential for professional practice and are strengthened through structured peer interaction and dialogue.^24-26^

Additionally, learning encompasses not only the formal curriculum, the structured, intentional content delivered through courses and assessments, but also the informal curriculum, which includes the interpersonal interactions, peer dynamics, and experiential learning that occur alongside planned instruction. Beyond these visible components lies the hidden curriculum, the implicit norms, values, power structures, and cultural expectations embedded within the educational environment. Together, these layers profoundly shape how learners interpret their roles, professional identity, and ethical responsibilities. Thus, it is essential to recognize that students learn not only the explicit subject matter but also the broader professional, social, and cultural context in which education takes place, influencing their behavior and development in ways that extend far beyond formal teaching.^27^

Implications for a research group, comprising PhD students and medical students doing undergraduate medical research projects, include cognitive gains, professional identity formation, research communication, skill acquisition, and scalability. Those who recently have learned from own mistakes can convey that information to their peers. Peer-led small groups activate prior knowledge and stimulate elaboration, mechanisms linked to better problem-solving in complex domains like neurosurgery.^28^ Near-peers, like senior PhD students, can scaffold literature appraisal, methodology choice, and data interpretation for medical degree-project students, while themselves, consolidating expertise through teaching.^11^ This observation is consistent with contemporary research demonstrating that students frequently describe peer-teaching activities as transformative experiences that strengthen confidence, professional development, and scholarly identity.^16^ Near-peer teaching appears at least as effective as expert teaching on knowledge and skills, with students often reporting greater comfort with near-peers, however, variability in teaching proficiency among peer tutors remains a concern. Mixed-methods evaluations also indicate comparable facilitation perceptions between near-peer and faculty tutors in problem-based learning (PBL), with qualitative gains in tutors’ professional identity as future medical educators. Structured peer feedback cycles on how to write abstracts, methods sections, and drafts can increase clarity and improve quality of the work for all involved parties.^22,24^ For undergraduate medical research students who conduct clinical skills simulations or data-collection tasks, PAL can be used for psychomotor rehearsal and formative appraisal.^14^

Design implications following Brierley et al include prioritizing PAL for practical skills teaching in the clinical phase, explicitly planning assessments to leverage retention benefits, and pairing PAL with faculty moderation to ensure theoretical domain coverage where PAL effects are weaker.^15^ Aligning session structure and matching of near-peers to learners’ stage and needs should be guided by the dimensions proposed by Ten Cate and Durning.^11^

Peer learning is not merely an adjunct but a high-impact, theoretically grounded strategy that advances cognitive, professional, and communicative capacities. Evidence from medical education and higher education shows that when designed with attention to congruence, training, structure, and assessment purpose, peer learning yields outcomes comparable to faculty-led teaching and, can provide sustained benefits beyond course completion by fostering identity formation, research literacy, and collaborative practice critical for developing clinician-scientists and scholarly teams.^15^ From an implementation perspective, structured peer-assisted learning may also be cost-effective compared with faculty-led instruction, as it leverages existing learner resources while maintaining comparable outcomes and reducing demands on faculty time and teaching infrastructure.^29^

## Discussion

The primary conclusion of this paper is that peer learning can be intentionally integrated into a neuroscience research environment through structured preparation, dynamic role allocation, near-peer coaching, and systematic debriefing. Contemporary evidence suggests that peer-assisted learning improves knowledge acquisition, practical skills development, learner engagement, and medium-term retention of learning outcomes.^15,17-19^ The present framework translates these established educational principles into a practical implementation strategy for mixed groups of doctoral and medical students working within a research-intensive environment.

A second key outcome is the proposed integration of evidence-based mechanisms identified by Smith et al (2024), namely preparation before collaborative work, dynamic role-shifting during task performance, and structured debriefing after completion of activities. These mechanisms appear particularly relevant because they support psychological safety, targeted feedback, progressive autonomy, and professional identity formation within longitudinal learning relationships.^5^ This emphasis on identity development aligns with contemporary scholarship suggesting that professional identity formation emerges through participation in authentic communities of practice rather than solely through acquisition of knowledge and skills.^21^

Concretely, the implementation plan includes instituting small mixed groups (PhD and medical students) that meet weekly and alternate between a research production focus (e.g., protocol design, analysis planning, scientific writing) and a clinical reasoning focus related to our patient population (e.g., persistent or recurrent pain after spine surgery). For preparation, the program will require brief pre-meeting submissions: a one-page problem representation for research meetings and a case stem with a prioritized differential and anticipated biases for clinical reasoning meetings. This step is designed to reduce extraneous load, prime prior knowledge, and allow near-peer coaches to tailor scaffolding.^28,30^

The implementation strategy is deliberately informed by the conceptual model described by Smith et al (2024). Their grounded theory study identified three recurring practices that characterized successful longitudinal near-peer learning environments: preparation before encounters, dynamic role negotiation during collaborative activities, and structured debriefing after encounters.^5^ The present implementation strategy also emphasizes continuity of participation. Stable peer groups, recurring role assignments, and longitudinal collaboration were selected because continuity of learner relationships has been associated with improved mentoring, stronger peer learning, and greater learner satisfaction.^6^

Preparation establishes shared expectations and identifies learner needs before collaborative work begins. Dynamic role-shifting enables senior learners to provide real-time scaffolding while preserving junior learner autonomy. Structured debriefing promotes reflection, feedback exchange, and calibration of future performance. Together, these mechanisms foster trust, social congruence, cognitive congruence, and psychological safety, all of which are associated with enhanced learner development and professional identity formation.^5,12^

Accordingly, the implementation model proposed in this paper directly operationalizes these three mechanisms through mandatory pre-session preparation, rotating peer-learning roles, and standardized reflective debriefings after each session.

During the session, dynamic role-shifting will be used to distribute cognitive work and responsibility. Rotating roles will include a junior Lead Presenter, a senior Near-Peer Coach who guides think-alouds and scaffolding, a Method/Skeptic (or Bias Spotter in clinical sessions) who challenges assumptions and calls diagnostic timeouts, and a Scribe who captures decisions and action items. This structure draws on social and cognitive congruence while operationalizing the role choreography highlighted by Smith et al.^5,12^ The research group leader will attend on a rotating cadence with senior colleagues to moderate, model expert heuristics, and ensure theoretical breadth and research ethics without displacing student agency.^11^

For debriefing, plans will include adopting a brief, standardized script: a plus-delta debriefing (what worked well and what should change), two commitments to action with timelines, and a reflective prompt (e.g., ‘what changed your mind?’). In the clinical stream, the debrief will also include a short error/near-miss analysis to surface pivotal reasoning moments and update illness scripts. These debriefs will be archived, building a shared memory for the group and enabling longitudinal assessment aligned with Kirkpatrick Levels 2–3.^31^

Psychological safety is a deliberate design goal. Plans will include introducing short rituals that signal inclusion and normalize uncertainty, rotating facilitation, visible agenda-setting, and explicit error framing (‘good catch’ language), in line with Edmondson’s and Clark’s work on team learning climates.^32-34^ To mitigate variability in peer coaching quality, the program will run two 90-minute coach workshops on facilitative feedback, scaffolding (worked examples, faded prompts), debriefing techniques, and bias mitigation, accompanied by lightweight observation with feedback during the first months.^7,9^

**Figure 1.**
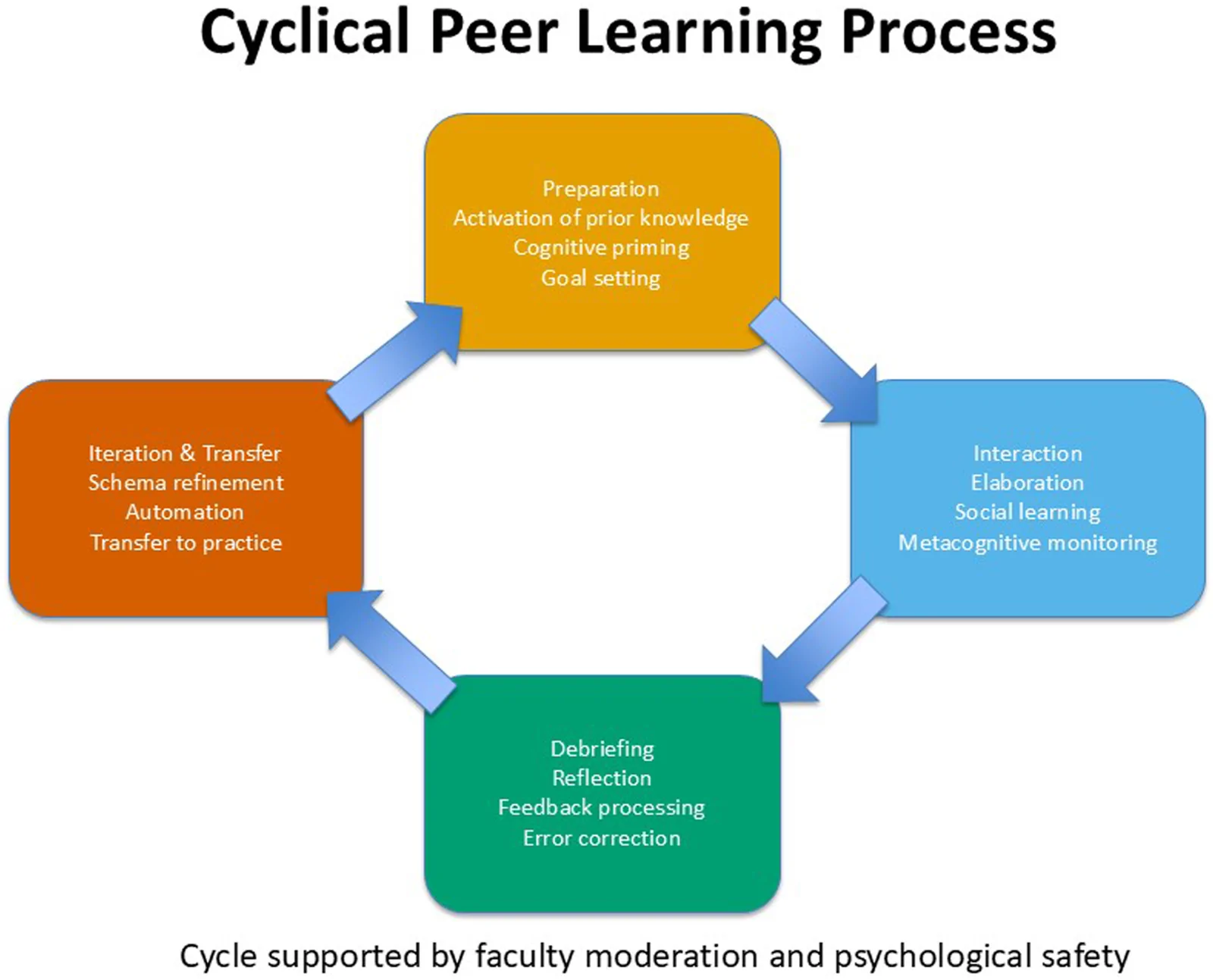
Cycle supported by faculty moderation and psychological safety

To enhance clarity and practical application, a conceptual figure illustrating the cyclical peer learning process and a structured implementation framework table are provided (Figure 1, Table 1).

**Table 1.** Structured Peer Learning Implementation Framework

Phase	Activity	Roles	Outputs	Evaluation metrics
Preparation	Pre-session tasks	All participants	Problem summaries, cases	Completion rates
Interaction	Discussion & role rotation	Presenter; coach; skeptic; scribe	Reasoning processes	Engagement; role adherence
Debrief	Reflection & feedback	Group	Action plans	Quality of reflection
Longitudinal	Program integration	Faculty and learners	Research outputs	Publications; milestones

## Framework and Evaluation Strategy

The Kirkpatrick Four-Level Evaluation Model was selected because it provides a pragmatic and widely recognized framework for evaluating educational interventions across progressively complex outcome domains, including learner reactions, knowledge and skill acquisition, behavioral transfer, and organizational outcomes. Although originally developed for workplace training, the framework has been extensively applied within health professions education because it allows alignment between educational design and intended outcomes.^31^

For the present peer learning model, Kirkpatrick’s framework is particularly suitable because the intervention aims to influence not only immediate learning outcomes but also longer-term scholarly and professional behaviors. Levels 1 and 2 capture learner experience, perceived psychological safety, research confidence, clinical reasoning development, and skill acquisition. Levels 3 and 4 evaluate whether these gains translate into sustained behaviors and meaningful academic outcomes, including scholarly productivity, project completion, and collaborative practice.

Contemporary educational evaluation literature suggests that outcome measures should be complemented by implementation-focused frameworks that examine how and why interventions succeed. Therefore, evaluation of the present model will additionally incorporate process indicators derived from implementation science, including participation rates, fidelity to role rotation procedures, quality of debriefing activities, and learner engagement. Combining outcome and process measures provides a more comprehensive understanding of intervention effectiveness.^35-37^

This approach is consistent with recent calls within health professions education to move beyond simple satisfaction measures and incorporate mixed-methods, longitudinal, and context-sensitive evaluation strategies that capture both learning outcomes and educational mechanisms.^38,39^

Assessment will be programmatic rather than event-based. At Level 1 (Reaction), one-minute post-session surveys will be used to assess perceived usefulness, psychological safety, and workload. At Level 2 (Learning), Calibrated rubrics will be used to assess growth in problem representation, clinical reasoning, and scientific writing over time. At Level 3 (Behavior), Transfer behaviors will be monitored through adherence to role rotations, completion of pre-brief activities, use of bias-checking procedures, and revision rates following feedback. Evaluation at Level 4 (Results) will include monitoring of undergraduate medical research projects completion, manuscript submissions, ethics approvals, and progress toward predefined milestones, as well as solicit faculty and clinical supervisor judgments of readiness and teamwork.^8,15,31^ Methodologically, Kirkpatrick Levels 1 and 2 are considerably easier to evaluate because they focus on learners’ immediate reactions and measurable gains in knowledge or skills, which can be captured through surveys, tests, or other direct assessment tools administered near the educational intervention. In contrast, Levels 3 and 4 require evidence of behavioral change in real-world settings and broader organizational or systemic outcomes, both of which are influenced by numerous contextual, interpersonal, and environmental variables. These higher-level evaluations often demand longitudinal follow-up, multi-source data collection, and more complex analytic strategies, making them substantially more challenging to assess reliably and to attribute directly to the intervention itself.

To strengthen the evaluation approach, the present framework extends beyond traditional outcome measures by incorporating qualitative and longitudinal methods. Qualitative data, including reflective logs and structured debrief analyses, will be used to capture changes in reasoning processes and learner engagement, complementing quantitative measures. In addition, longitudinal tracking will be used to assess behavioral change and sustained outcomes, including development of clinical reasoning, research productivity, and progression toward academic milestones. This aligns with contemporary evaluation approaches recognizing that higher level outcomes (Kirkpatrick Levels 3–4) require multi source data and longer time horizons.

The intention is to put in place practical guardrails. To address time pressure and logistics, meetings will have fixed time slots, hybrid participation standards, and concise pre-brief templates. To prevent over-reliance on senior voices, role rotations will be enforced to ensure that juniors regularly lead discussions. To avoid superficial debriefs, the debrief script will include explicit commitments to action and scheduled follow-ups. Finally, to prevent cognitive overload in more junior members, task complexity will be calibrated to progressively fade scaffolds as competence grows.^30^

Despite its benefits, peer learning is not without risks. Prior literature has identified challenges including variability in tutor quality, unequal participation, and the potential reinforcement of misconceptions when peer interactions are insufficiently structured.^14^ Recent studies further highlight risks of cognitive overload and imbalance in participation within peer groups. These risks are mitigated in the present model through structured role allocation, faculty moderation, and targeted tutor training, which are key design principles for effective peer assisted learning environments.^4^

In sum, the intention is to preserve the strengths of an existing peer learning culture while making the process more systematic, transparent, and developmentally aligned.

By embedding near-peer mechanisms, preparation, dynamic role-shifting, structured debriefing, and continuity of learner relationships within psychologically safe routines, it is anticipated to see more consistent gains in reasoning quality, research outputs, and professional identity formation across the group.^5,6,20^

## Summary

The congruence between near-peer tutors and learners situates guidance appropriately within the learners’ developmental zone, thereby optimizing cognitive load and communication.^1,5,12^ Modeling of expert-like strategies by slightly more advanced peers, reinforced through feedback loops, strengthens self-efficacy and persistence.^2,7,40^ Peer assessment mechanisms provide elaborated, dialogic feedback, which supports evaluative judgment and deeper learning.^9,22,24^

Three design levers stand out: (1) Tutor training focused on feedback specificity, questioning techniques, and error framing to reduce variability in teaching quality.^7,9^ (2) Structure and matching, clear roles, session scripts, and alignment of educational distance, enhance effectiveness.^8,11^ (3) Faculty moderation provides targeted oversight to ensure theoretical domain coverage and addresses areas where PAL effects are weaker, particularly in abstract theoretical knowledge.^4,13,15^

Planned timing of assessments and booster sessions capitalizes on PAL’s retention benefits in practical skills.^15^ Transfer is strengthened by embedding PL practices into routine scholarly activities (journal clubs, writing circles), aligning with communities of practice.^20^ Moreover, assessments must be explicitly aligned with the intended learning objectives to ensure that what is being measured genuinely reflects the knowledge, skills, and professional capacities that the curriculum aims to develop. Within this framework, peer learning should be understood not as a standalone pedagogical solution but as one contributory activity that supports constructive alignment, where learning activities, assessment tasks, and outcomes are deliberately coordinated to reinforce one another. In Biggs’ model, constructive alignment requires that every instructional component works synergistically toward the same goals.^41^ Thus, peer learning can enhance engagement, feedback literacy, and collaborative competence, but it must be embedded within a broader, coherently designed educational strategy rather than functioning as a complete teaching method on its own.

## Actions

Plans will include: (a) offering short tutor-development workshops on feedback typologies, scaffolding, and inclusivity; (b) implementing structured peer-feedback with calibrated rubrics and exemplars for abstracts, methods, and visualizations, including dialogic cycles and tracked revisions; (c) emphasizing PAL for practical skills in the clinical phase with spaced assessments and simulation checklists; (d) maintaining faculty moderation to ensure theoretical breadth, ethical and statistical rigor, and alignment with assessment standards; and (e) institutionalizing peer-led journal clubs, method clinics, and writing circles to nurture educator identities and a sustainable scholarly culture.^7-9,11,14,15,22,24^

## Conclusion

Peer learning, when designed with attention to congruence, training, structure, and assessment purpose, remains a high-impact strategy for advanced medical and doctoral education. By intentionally shifting to routines that emphasize near-peer mechanisms, psychological safety, and programmatic evaluation, Plans will include delivering measurable gains in reasoning, productivity, and team culture consistent with the broader evidence base. This approach provides a robust pathway for developing clinician-scientists and scholarly teams in neurosurgery while supporting professional identity formation through structured participation in collaborative scholarly practice.^5,15,21^ This manuscript is therefore positioned as a reflective and practical advances contribution, integrating contemporary empirical evidence with a structured implementation model to support clinician educators in designing peer learning environments.
